# Canonical and non-canonical functions of p53 isoforms: potentiating the complexity of tumor development and therapy resistance

**DOI:** 10.1038/s41419-024-06783-7

**Published:** 2024-06-12

**Authors:** Yitian Guo, Hang Wu, Lisa Wiesmüller, Ming Chen

**Affiliations:** 1 Department of Urology, Zhongda Hospital Southeast University, Nanjing, China; 2 Department of Rehabilitation Medicine, Zhongda Hospital Southeast University, Nanjing, China; 3 Department of Obstetrics and Gynecology, Ulm University, Ulm, Germany

**Keywords:** Mechanisms of disease, Tumour-suppressor proteins

## Abstract

Full-length p53 (p53α) plays a pivotal role in maintaining genomic integrity and preventing tumor development. Over the years, p53 was found to exist in various isoforms, which are generated through alternative splicing, alternative initiation of translation, and internal ribosome entry site. p53 isoforms, either C-terminally altered or N-terminally truncated, exhibit distinct biological roles compared to p53α, and have significant implications for tumor development and therapy resistance. Due to a lack of part and/or complete C- or N-terminal domains, ectopic expression of some p53 isoforms failed to induce expression of canonical transcriptional targets of p53α like *CDKN1A* or *MDM2*, even though they may bind their promoters. Yet, p53 isoforms like Δ40p53α still activate subsets of targets including *MDM2* and *BAX*. Furthermore, certain p53 isoforms transactivate even novel targets compared to p53α. More recently, non-canonical functions of p53α in DNA repair and of different isoforms in DNA replication unrelated to transcriptional activities were discovered, amplifying the potential of p53 as a master regulator of physiological and tumor suppressor functions in human cells. Both regarding canonical and non-canonical functions, alternative p53 isoforms frequently exert dominant negative effects on p53α and its partners, which is modified by the relative isoform levels. Underlying mechanisms include hetero-oligomerization, changes in subcellular localization, and aggregation. These processes ultimately influence the net activities of p53α and give rise to diverse cellular outcomes. Biological roles of p53 isoforms have implications for tumor development and cancer therapy resistance. Dysregulated expression of isoforms has been observed in various cancer types and is associated with different clinical outcomes. In conclusion, p53 isoforms have expanded our understanding of the complex regulatory network involving p53 in tumors. Unraveling the mechanisms underlying the biological roles of p53 isoforms provides new avenues for studies aiming at a better understanding of tumor development and developing therapeutic interventions to overcome resistance.

## Facts

- p53 isoforms modulate canonical functions such as in cell cycle control and apoptosis as well as non-canonical functions in DNA repair and replication.
- p53 isoforms exert biological roles via p53α-independent transcriptional activities (p53β/γ and Δ40p53) or via hetero-oligomerization with p53α impacting on p53α activities (Δ40/133/160p53).
- The co-existence of different p53 isoforms potentiates the complexity of their biological functions.
- Tumor-specific expression profiles of p53 isoforms are of interest for diagnostic and prognostic implications, paving the way for tailored treatment strategies.

## Open questions

Do alternative p53 isoforms impact on

- Cancer stemness, epithelial-mesenchymal transition (EMT), metastasis and chemoresistance via non-canonical functions in DNA repair and replication?
- The spectrum and penetrance of hereditary tumors in Li-Fraumeni patients with germline *TP53* mutations?

## Introduction

Human *Tumor Protein 53* (*TP53*) gene, situated on chromosome 17p13.1 [1], comprises 13 exons, including 11 constitutive exons and 2 alternatively spliced exons [2]. Despite its identification already 40 years ago, ongoing research continues to unveil novel functions, activities, and interactions associated with this gene.

### The identification of p53 isoforms

*TP53* splice variants were initially identified in the 1980s [3, 4], but their presence in different species and their biological and clinical importance were only established around 15 years later [2]. Through the utilization of various mechanisms like different promoters, alternative splicing, and internal ribosome entry site (IRES), *TP53* generates 12 distinct isoforms including full length p53α (also known as p53 Wild Type or p53WT) [5, 6].

**Fig. 1:**
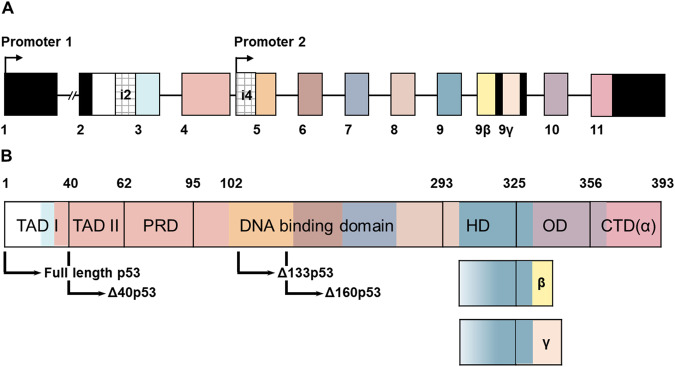
*TP53* gene and p53 domains [2, 8, 37, 48, 65, 189]. **A** The *TP53* gene consists of canonical exons (represented by colored boxes) that encode different structurally and functionally defined domains of the p53 protein. Transcription from promoter 1 generates an mRNA transcript capable of translating into full-length p53 (FLp53) or ∆40p53 isoforms. ∆40p53 isoforms are only translated when intron 2 is present in the mRNA transcript. On the other hand, transcription from promoter 2 produces an mRNA transcript that codes for ∆133p53 or ∆160p53 isoforms through differential translation initiation. Alternative splicing of exon 9 modulates the production of C-terminally variant isoforms of p53 (α, β, and γ). **B** p53 protein consists of six distinct domains that are encoded by different exons of the *TP53* gene, as depicted in (**A**) (with exons and coding domains shown in matching colors). Within the transactivation domain (TAD) and DNA-binding domain (DBD) arrows indicate the starting points of specific isoforms. On the bottom right, colored boxes schematically outline the two C-terminally altered isoforms: β and γ.

As shown in Fig. 1A, canonical *TP53* transcription starts from the promoter 1 while non-canonical transcription starts from promoter 2 [7]. In human cells, the transcript from promoter 1 produces p53α, alternative splicing produces variants that contain intron 2 and intron 9. Alternative splicing of intron 9 leads to the creation of mRNA variants containing exons 9β or 9γ, resulting in the β and γ subtypes. Both exon 9β and 9γ contain stop codons, causing exons 10 and 11 to remain untranslated in *TP53* β and γ mRNA variants. On the other hand, the α isoform includes all exons [2, 8]. p53α binds promoter 2 (P2) upon DNA damage signals (camptothecin, doxorubicin), which induces Δ133p53/Δ113p53 in human tumor cells and zebrafish, respectively [7, 9, 10]. Others observed that not only in human cancer cells but also in human induced pluripotent stem cells (iPSCs), P2 transcription was induced by DNA damage (X-ray) as well as by cell ageing through prolonged passaging of cells, whereby site-specific CpG demethylation events in exon 5 were found to be spatially and temporally associated with transcription from the adjacent intronic P2 [11].

### Different domains of p53 isoforms

p53 protein typically exists as a tetramer, composed of four monomers, with canonical functions as a transcriptionally transactivating factor [12–16]. The assembly of p53 tetramers, also called self-association, leads to DNA looping which links separated p53 DNA binding sites to increase p53’s concentration at one response element (RE) and/or activate additional REs [17]. Within the p53 monomer the following domains have been defined based on 3-dimensional structures and biochemical functions (Fig. 1B).

The intrinsically disordered region (IDR) within the N-terminal region of p53 has emerged as a crucial player in signaling cascades [18]. Despite having low affinity, the IDR enables highly specific interactions with other proteins [19]. TAD (transactivation domain), situated within the IDR, interacts with various proteins mediating and/or regulating p53’s functions including factors of the basal transcription machinery [20–24], the E3 ligase murine double minute 2 (MDM2) [25–28], or histone acetyltransferase p300-cAMP-response element binding protein (CREB) binding protein [29–31], as well as DNA repair and replication proteins including DNA polymerase β, α and ɩ, the single-strand binding protein Replication protein A (RPA) and the processivity factor Proliferating Cell Nuclear Antigen (PCNA) in DNA replication [32–36]. TAD can be divided into TAD I and TAD II (Fig. 1B) [37]. TAD I plays a critical role in p53-dependent transactivation, DNA damage-induced G1 cell cycle arrest, and apoptosis. Its functional importance outweighs that of TAD II in these processes [38] though TAD I and thereby full transcriptional transactivation is dispensable for tumor suppressive signaling pathways of p53 [2, 39, 40]. p53 and MDM2 establish an autoregulatory negative feedback loop, maintaining low p53 levels in unstressed cells. MDM2-p53 TAD interaction hinders the transcriptional ability of p53 [26–28, 41].

The Proline-Rich Domain (PRD) connects the TAD with the DNA-binding domain (DBD) [8]. This region contains five polyproline (PXXP) motifs, serving as binding sites for Src-Homology-3 domains that facilitate protein-protein interactions involved in signal transduction [42, 43]. Additionally, by undergoing proline isomerization, PRD alters 3-dimensional protein structure, adjusting the direction and angle of functionally interacting domains [44]. The PRD is necessary for growth inhibition and apoptosis triggered by p53 [45] and functioning as a spacer or scaffold module necessary for tumor suppression capabilities of p53 [2, 44, 46, 47].

DBD is the core domain of p53 and mediates the interaction between p53 and DNA. Within the DBD, there are several highly conserved histidines and cysteines that facilitate coordination with Zn^2+^ or Mg^2+^ ions, enabling proper p53 conformation and DNA binding ability [48–50]. Furthermore, the interaction between the p53 DBD and its N-terminal region contributes to the stability of p53 as a tetramer [51]. Most oncogenic mutations within the DBD result in conformational changes and/or alterations in p53’s ability to bind to target p53-specific DNA sequences. The large number of oncogenic mutations found in this domain underscores its crucial role in regulating tumor suppression [13, 15, 49, 52–54]. As seen in Fig. 1B, Δ40p53 isoforms possess the full-length DBD, while Δ133p53 and Δ160p53 isoforms lack significant portions of it. In particular, Δ160p53 lacks the entire first conserved cysteine motif, and is severely compromised in DNA binding, while Δ133p53 dimers still show some proficiency in binding p53-specific DNA elements [55]. Of note, the central domain with metal complexing capacity is also critical for p53´s activities in binding to RAD51 [56], recognizing DNA junctions, particularly comprising mispairings [57–59] and degrading such DNA structures exonucleolytically [60–62]. The integrity of the DBD therefore also is a prerequisite for p53´s non-canonical, i.e. transcription-independent functions in restricting low-fidelity homologous recombination and bypass of DNA replication impediments [36, 63, 64]. So far, such non-canonical functions in DNA repair have been investigated mostly for the p53α isoform.

The Hinge Domain (HD) links the DBD and Oligomerization Domain (OD) and comprises the Nuclear Localization Signal (NLS) [65]. HD enables p53 to enter the nucleus and provides structural flexibility for binding to REs [66]. Germline mutations of p53 in the HD, such as p53R306P, result in loss of transcriptional activation of p53 target genes like *BCL2-Associated X* (*BAX)* [67]. Moreover, p53 without HD is unable to recognize the consensus sequences, indicating that HD contributes to allosteric regulation of DNA binding [2, 67].

The OD is crucial for the formation of p53 tetramers and houses the nuclear export signal (NES). Tetramerization conceals the NES, keeping p53 within the nucleus to regulate target gene expression [68]. The OD assists in DNA deformation and facilitates stable DBD-DNA binding [69] such as when mediating apoptosis and cell cycle arrest [70]. Oligomerization via the OD equally promotes p53´s interactions with DNA junction structures to execute DNA recombination and replication control [36, 71].

The Carboxy-terminal domain (CTD) regulates the structure and function of the protein itself [72] and contains multiple post-translational modification sites which modulate protein degradation, tetramerization, transactivation and protein interactions [73, 74]. The extreme CTD is rich in positively charged amino acids like arginines, histidines, and lysines, enabling p53 to bind to negatively charged nucleic acids such as RNA and DNA indiscriminately [75], recognize DNA lesions and mismatches and promote DNA annealing [76–78]. Numerous proteins bind to the CTD, which explains why many p53 missense mutants still retain biochemical and biological activities [2]. Additionally, the nonspecific DNA binding capacity of the CTD allows p53 to diffuse along the DNA linearly or to transfer itself to another DNA molecule [79].

## The biological roles of p53 isoforms and underlying mechanisms

p53 protein, with its diverse isoforms, emerges as a multifaceted player in maintaining cellular homeostasis and safeguarding the genome. In this section, we delve into the biological roles of the different p53 isoforms to unravel their intricate contributions to cellular processes. Table 1 summarizes these biological effects and the biochemical activities potentially underlying these functions.

**Table 1:** Classification of p53 isoforms according to their biological effects in different cell types/animals.

Functions	p53 isoforms	Cell lines/Models	Activity	Effect	Ref.
Cell cycle and senescence/ aging	p53β	Human fibroblast lines CD8+ T lymphocytes Hutchinson-Gilford progeria syndrome (HGPS) derived fibroblasts	• p53β collaborates with p53α to upregulate p21 expression	↑senescence	[102, 103, 174]
	Δ40p53	Transgenic mice	• collaborates with p53α to transactivate Cyclin dependent kinase inhibitor 1A (Cdkn1a), Mdm2, and Insulin-like growth factor-binding protein 3 (Igfbp-3)	↑senescence and aging	[136, 175]
	Δ40p53	Transgenic mice	• collaborates with p66Shc to repress oxidative stress-specific gene set	↑cell-cycle arrest, senescence, and aging	[176]
	Δ40p53α	Human H1299 and HCT116 Human H1299 Human A375 melanoma cells	• upregulates miR-4671-5p to inhibit *N-Sulfoglucosamine Sulfohydrolase* (*SGSH)* expression • Δ40p53α alone failed to induce p21 • Exogenous Δ40p53α increases binding of p53α to *CDKN1A* promoter but suppresses *CDKN1A* expression	↑cell-cycle arrest no effect on cell-cycle	[84, 137, 138]
	Δ40p53α	Human fibroblasts	• elevated Δ40p53α/p53α ratio decreases p21 expression	↓cell-cycle arrest	[82]
	Δ122p53α (mouse ortholog of human Δ133p53α)	Transgenic mice	• represses transactivation of *Mdm2* and *Cdkn1a* by p53α	↓cell-cycle arrest	[97]
	Δ133p53α	Human Aortic Smooth Muscle Cells (HAMSCs)	• represses p21 expression via upregulating Krüppel-like factor 5 (KLF5)	↓cell-cycle arrest	[100]
	Δ133p53α	Human neonatal foreskin and normal prostate tissue	• represses *CDKN1A* expression • upregulates Human telomerase reverse transcriptase (TERT) expression	↓senescence ↑proliferation	[98]
	Δ133p53α	Human U2OS cells	• Δ133p53α increased p21 mRNA expression without altering protein expression	↓cell-cycle arrest	[7]
	Δ133p53	Human fibroblast lines CD8+ T lymphocytes Hutchinson-Gilford progeria syndrome (HGPS) derived fibroblasts	• Δ133p53 represses expression of p53 target genes incl. *CDKN1A* and *miR-34a*	↓senescence	[102, 103, 174]
	Δ246p53	Human HEK293T, LN229, A549, HT1080, HCT116, SW480 and MCF7 lines	• upregulates *CDKN1A* by binding to p53α	↑senescence	[177]
Apoptosis	p53β and p53γ p53β	Human MCF7 and H1299 lines Human 786-O cells and CAKi-1 cells	• upregulates *BAX* by enhanced transcriptional activity of p53α	↑apoptosis ↑apoptosis	[93, 94] [120]
	Δ40p53α	Human Saos 2 cells	• induces apoptosis related genes: Nucleolysin TIAR (TIAL1) and Apoptosis-stimulating protein of p53 2 (ASPP2) which are not induced by p53α	↑apoptosis	[86]
	Δ40p53α	Human MCF-7 and ZR75-1 cells	• reduced/elevated level Δ40p53α/53α ratio upregulates/represses *BAX*, *NADPH Oxidase Activator 1* (*NOXA)*, *P53 Up-Regulated Modulator Of Apoptosis* (*PUMA)* transcription	↑/↓apoptosis	[87]
	Δ40p53α	Human H1299 cells	• transcriptionally upregulates *BAX*	↑apoptosis	[84]
	Δ40p53α	Human A375 melanoma cells	• upregulates *p53-induced protein with a death domain* (*PIDD*) by target gene-specifically enhancing transcriptional activity of p53α	↑apoptosis	[138]
	Δ113p53 (zebrafish ortholog of human Δ133p53)	Zebrafish	• de-represses *bcl-2-like 1* (*bcl2L*) expression by target-specific modulation of p53α´s transcriptional activity	↓apoptosis	[9, 178]
	Δ122p53α	Transgenic mice	• upregulates Baculoviral repeat-containing 5 (Birc5) and TNF receptor-associated factor 1 (Traf1) • no transactivation of *Bax* or *Puma*	↓apoptosis	[97]
	Δ133p53α	Human neonatal foreskin and normal prostate tissue	• reduces *PUMA*, *NOXA*, *BAX* expression by weakening transcriptional activity of p53α	↓apoptosis	[94, 98]
	Δ133p53α	Human vascular smooth muscle cell	• stimulates serine/arginine-rich splicing factor 1(SRSF1)–B-cell lymphoma-extra large (Bcl-xL) signal	↓apoptosis	[100]
	Δ133p53α	Human U2OS cells	• directly interacts with p53α to induce BCL-2	↓apoptosis	[7]
	Δ133p53β	Human HCT116, SW480, LoVo, SW620, Colo205 cells	• interacts with and inhibits anti-apoptotic Ras Homolog Family Member B (RhoB)	↑apoptosis	[168]
DNA replication/ repair	Δ40p53α	Human MCF-7 and ZR75-1 cells	• elevated Δ40p53α/53α ratio facilitates the transcription specifically of *RAD51* • doxorubicin treatment led to the formation of nuclear p53α-RAD51-BRCA1 but not to Δ40p53α-RAD51-BRCA1 complexes	↑DNA repair	[87]
	Δ133p53	HGPS fibroblasts	• increases expression of *RAD51* via de-repression of p53α target E2F Transcription Factor 1 (*E2F1)*	↑DNA repair	[103]
	Δ133p53/ Δ113p53	Human QSG-7701 cells/ Zebrafish	• upregulates the transcription of rad51, DNA ligase 4 (lig4), X-Ray Repair Cross Complementing 4 (xrcc4), rad52, rad54, RecQ Like Helicase 4 (recq4) and Meiotic Recombination 11 (mre11)	↑DNA repair	[90]
	Δ122p53α	Transgenic mice	• upregulates Valosin-containing protein (VCP) expression	↑DNA repair	[140]
	Δ133p53α	Human Saos 2, HCT116, H1299 cells	• acts synergistically with p73 to promote the expression of RAD51, LIG4 and RAD52	↑DNA repair	[91]
	Δ133p53α and Δ160p53α	Saos 2, K562, human hematopoietic stem and progenitor cells	• inhibit p53α/POLɩ-mediated DNA damage tolerance pathway	↑DNA replication	[81]
Inflammatory response	Δ122p53	Transgenic mice	• upregulates IF-6 and Interferon-gamma (IFN-γ) • stimulates NF-кB signaling to increase Tumor necrosis factor (TNF)-α. • upregulates chemokine and cytokine signaling including Signal Transducer And Activator Of Transcription 1 (Stat1), Transcription factor jun-B (Junb).	↑inflammatory response	[97, 140–142, 179]
Pluripotency	Δ40p53α	Human glioblastoma tissues, ESCs, subventricular (SVZ) progenitor cells	• Expression profile in glioblastoma resembles pluripotent ESCs. • Differentiation decreases Δ40p53α expression in SVZ progenitor cells.	↑pluripotency	[128]
	Δ40p53α	129/SvJ mouse ESCs	• blocks repression of *Nanog* and *IGF-1R* by p53α	↑pluripotency	[95, 96, 104]
	Δ133p53β	Human MCF-7 cells	• upregulates the expression of Sex determining region Y box 2 (SOX2), Octamer-binding transcription factor 3/4 (OCT3/4), NANOG	↑pluripotency	[98, 180, 181]
Cellular invasion	Δ40p53α	Human MCF-7 and ZR75-1 cells	• regulate genes correlated to migration, invasion	↓migration and invasion	[135]
	Δ133p53β	Human MDA-MB-231, D3H2LN, MCF7, LoVo, SW480, SW620, Colo205, HCT116 cells	• associated with E-cadherin and β1-integrin downregulation	↑migration and invasion	[150]
	Δ122p53	Transgenic mice	• upregulates Integrin subunit beta 7 (Itgb7) and Vascular cell adhesion protein 1 (Vcam1).	↑adhesion	[97]
	Δ122p53/ Δ133p53	Transgenic mice, human HCT116 cells	• activates inflammatory Janus kinase (JAK)-STAT and Ras Homolog Family Member A (RhoA)- Rho-associated protein kinase (ROCK) signaling in an IL-6-dependent manner	↑invasion	[143]
	Δ160p53α	Human H1299, MCF10A cells	• Mutant *TP53* requires Δ160p53α for pro-oncogenic potential	↑adhesion and invasion	[92]
	Δ160p53α/β	Human H1299 cells	• can be imported to nucleus and bind chromatin	↑proliferation and migration	[156]

Alterations in protein/gene expression/activity are presented as a function of p53 isoform levels.

### p53 isoforms and biochemical activities

#### p53α-independent activities of alternative p53 isoforms

**Fig. 2:**
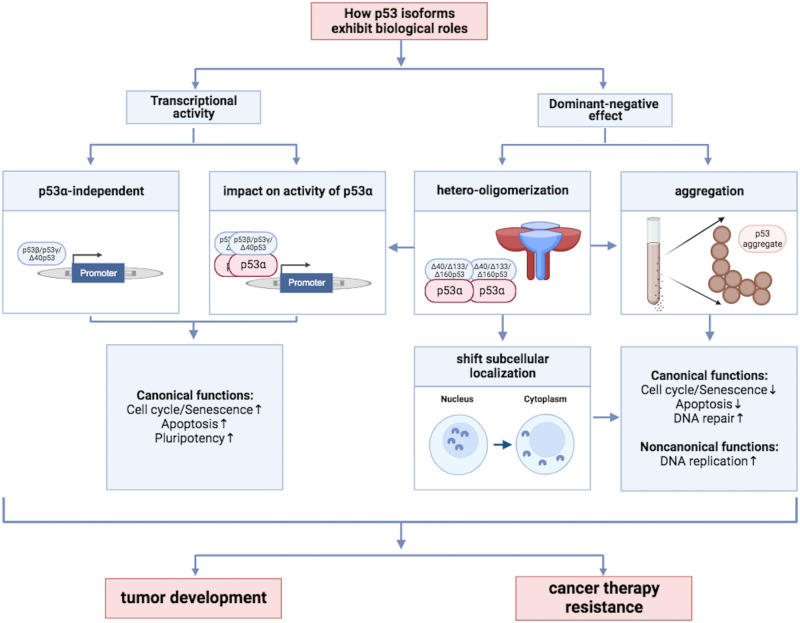
How alternative p53 isoforms exhibit biological functions.

Some p53 isoforms exert their cellular roles independently of p53α (Fig. 2). Ectopically expressed p53β and p53γ were demonstrated to be incapable of activating the p53α target genes *DNA Damage Inducible 1 Homolog 2 (DDI2), Arginase 2 (ARG2), CDKN1A, E2F Transcription Factor 7 (E2F7), Serpin Family E Member 1 (SERPINE1), Tumor Protein P53 Inducible Nuclear Protein 1 (TP53INP1)* or *TP73* through promoter reporter assays in H1299 cells [80]. In K562 and Saos 2, ectopic expression of alternative p53 isoforms in general failed to induce MDM2 or p21 [81]. Previous studies have shown that Δ40p53α shows less binding affinity to *Mdm2* and *Cdkn1a* promoter compared to p53α in Balb/c 10.1 fibroblasts [82] and mediates no measurable p21 protein induction in H1299 and Saos 2 cells [81, 83]. Though defective in some canonical transcriptional activities of p53α, Δ40p53α binds and activates genes including *BAX* due to retention of its TAD II [84]. Furthermore, Δ40p53α transactivates a different set of p53-responsive genes than p53α [85], in particular, several apoptosis related genes: *TIAL1* and *ASPP2* which are not induced by p53α [86].

Regarding a direct, i.e. transcription-independent function of an alternative p53 isoform in DNA repair, the only piece of information accumulated so far has been that Δ40p53α has lost the capability to form complexes with the key homologous recombination factors RAD51 and BRCA1 in the nucleus of doxorubicin-treated cells [87]. Yet it is unclear why TAD I would be required for formation of these complexes, as the physical interaction of p53 with RAD51 requires central [56] and with BRCA1 C-terminal binding sites on p53 [88]. Therefore, either p53 recognizes the same lesions on DNA as RAD51 and BRCA1 and/or even cooperates with RAD51 and BRCA1 in fork remodeling at DNA replication barriers which requires the N-terminal POLɩ binding site [36, 89]. If the latter were true, this observation would be more relevant for non-canonical activities of p53 in DNA replication than DNA repair (see Chapter 2.2).

The Δ133p53α isoform responds to γ radiation and binds to novel p53 REs in the promoters of DNA double strand break (DSB) repair genes (including *RAD51*, *RAD52* and *LIG4*), which upregulates the transcription of these genes independently of p53α [90]. In H1299 cells which are p53α negative, the overexpression of Δ133p53α augments DSB repair, whereby depletion of p73 revealed dependency on this p53 family member. Chromatin immunoprecipitation experiments demonstrated that the isoform Δ133p53α and p73 collaborate to upregulate the expression of DSB repair genes including *RAD51*, *RAD52* and *LIG4* through synergistic occupancy of the corresponding REs independently of p53α [91].

Δ160p53 was reported to mediate molecular functions of oncogenic p53 gain of function mutants towards proliferation and invasion of tumor cells [92]. Accordingly, it was described that the Δ160p53α isoform is commonly involved in the gain of function phenotype of mutant p53 proteins like p53(273H), p53(175H) or p53(248W). Stable and transient expression of p53(273H) as well as some endogenously mutant p53 expressing cell lines exhibit Δ160p53α expression [92]. Moreover, the authors causally linked the gain of function activities of the p53 mutants with the expression of Δ160p53α revealing the pro-oncogenic property of Δ160p53α [92], which might also be due to fork stalling caused by Δ160p53α independently of p53α upon DNA damage [81].

#### Modulation of transcriptional activities of p53α by alternative p53 isoforms

Alternative p53 isoforms can alter the transcriptional activities of p53α (Fig. 2). Though lacking the typical OD partially, p53β can still form a complex with p53α at the *BAX* promoter in an incompletely understood fashion and augment p53α´s intrinsic transactivation activity specifically on the *BAX* promoter [93, 94].

Δ40p53α and Δ133p53α isoforms retain the full OD for hetero-oligomerization with p53α or other α isoforms. Forming part of such hetero-oligomeric complexes Δ40p53α, lacking the N-terminal MDM2-binding site, protects complexed p53α from degradation and shifts transcriptional transactivation activity from *CDKN1A* to *BAX* [2, 83, 84]. In ESCs from transgenic mice, the Δ40p53α/p53α complex controls the transition from pluripotency to differentiation, whereby elevated Δ40p53α levels prolong pluripotency by upregulating Nanog as well as Insulin-like growth factor 1 receptor (IGF-1R) [95]. Thereby, Δ40p53α sequesters p53α within dimers and tetramers in the cytoplasm diminishing binding of p53α to *Cdkn1a*, *Nanog*, and *IGF-1R* significantly reducing transcriptional activity of p53α on *Cdkn1a* and de-repressing transcriptional activity of p53α on *Nanog*. In human breast cancer (BRCa), Δ40p53α expression was also reported to associate with upregulation of stemness genes, confirming a species-independent role of this isoform in inducing stemness [96].

In transgenic mice, Δ122p53α (mouse ortholog of human Δ133p53α) decreases transactivation of *Mdm2* and *Cdkn1a* by p53α [97]. In human neonatal foreskin and normal prostate tissue, Δ133p53α similarly reduces expression of p53α targets *CDKN1A*, *PUMA*, *NOXA* and *BAX* [98].

In conclusion, while C-terminally altered p53 isoforms cooperate with p53α during transcription, Δ133p53α exerts an inhibitory effect and Δ40p53α modulates transcription, i.e. promotes and mitigates p53α activities depending on the particular target gene. The impact of alternative isoforms, Δ40p53α in particular, on p53α transcriptional activities can vary depending on the cellular context, such as cell type, cellular differentiation program, availability of specific cofactors and the composition of the specific isoforms.

### Dominant negative effects of alternative p53 isoforms

A dominant negative effect of oncogenic mutant p53 exerted via hetero-oligomerization was initially reported in 1991 [99]. More than a decade later, Δ133p53α was proven to exert a dominant negative effect on transcriptional activities of p53α towards apoptosis induction, concomitantly unleashing p53α-repressed genes in DNA repair and cell cycle progression [9, 97, 98, 100, 94, 101]. In human fibroblasts from patients with the premature aging disorder Hutchinson-Gilford Progeria Syndrome (HGPS), hetero-oligomerization as the basis of the dominant negative effect was proven by co-immunoprecipitation of p53α with FLAG-labeled Δ133p53α. Further evidence from these HGPS, but also normal fibroblasts showed that Δ133p53α blocks p53α-mediated induction of *CDKN1A* mRNA and miR-34a, and thereby ultimately replicative senescence [102, 103]. In the same cell model Δ133p53α also induced RAD51 via de-repression of the p53α target *E2F1*. p53α and Δ133p53α induction and subsequent Δ133p53α-dependent upregulation of RAD51 was also observed during induced pluripotent stem cells reprogramming, and may serve to ensure DNA repair and genome stability in the absence of apoptosis in these primitive cells [104]. Interestingly, in different cell lines genotoxic treatment with doxorubicin was shown to induce Δ133p53α by p53α-mediated transactivation of the internal *TP53* promoter. In this scenario Δ133p53α, again co-precipitating with p53α, dampens p53α-dependent apoptosis and G1 but not G2 cell cycle arrest as part of a feedback loop modulating the cellular response to DNA damage [7]. Altogether, Δ133p53α dominant negatively affects transcriptional programs towards the canonical functions of p53α in cell cycle control, senescence, apoptosis and DNA repair.

The idea of a transcription-independent role of p53 in DNA replication emerged already in 1993, when p53 was found to directly bind to RPA and to stimulate Bovine Papilloma Virus 1 (BPV-1) replication [32, 105]. Multiple pieces of evidence, notably also the discovery of p53´s intrinsic 3´-5´exonuclease activity acting both in nuclei and the mitochondria [60, 106], established the concept that p53 executes non-canonical functions in DNA replication processes [107–109]. While the mechanistic details in mitochondria are still underexplored [110, 111], the precise role of p53α in nuclear DNA replication has been unveiled through the discovery of the p53α-POLι dependent DNA damage tolerance (DDT) pathway: When replication forks encounter replication barriers such as crosslinks, p53α collaborates with the highly error-prone POLι in a process known as idling events, i.e. iterative insertion and exonucleolytic removal of misincorporated nucleotides. This cooperative mechanism slows down DNA synthesis and enables bypass of the barrier through the involvement of the fork reversal enzymes HLTF and ZRANB3 [64]. A major clue leading to this model came from the separation-of-function mutant p53(H115N), fully functional in transcription but lacking exonuclease activity [112], and inactive in this DDT pathway. This pathway serves to safeguard DNA replication in human stem cells, including cancer stem cell-like cells, by preventing fast and error-prone bypass mechanisms [64, 113].

In crosslinker treated K562 and Saos 2 cells, human cell types devoid of p53α, expression of each of five alternative p53 isoforms (p53β, p53γ, Δ40p53α, Δ133p53α, Δ160p53α) indicated loss-of-function in this DDT pathway [81]. This phenotype can be explained by loss of critical domains necessary for oligomerization (p53β, p53γ), PCNA and POLι binding (Δ40p53α, Δ133p53α, Δ160p53α), RPA binding and exonucleolytic activity (Δ133p53α, Δ160p53α) [36, 64], respectively. Both co-expression of p53α and of one of the alternative isoforms (K562, Saos 2) as well as selective depletion of specific isoforms (HSPCs) revealed a dominant negative effect of Δ133p53α and Δ160p53α on p53α regardless of the treatment conditions [81]. Of interest with regard to the underlying mechanism, Δ133p53α and Δ160p53α expressed in unperturbed K562 and Saos 2 cells caused severe stalling of replication. From this, the dominant negative effect exerted by Δ133p53α and Δ160p53α may be due to the formation of non-productive hetero-oligomers with p53α and/or occupation of DNA replication sites through DNA and protein interactions via the residual DBD and CTD. Therefore, dominant negative effects of severely N-terminally truncated p53 isoforms are critical mechanisms affecting both canonical and non-canonical functions of p53α (Fig. 2).

The composition of p53 isoform hetero-oligomers determining the biological activities is influenced by the relative levels of the different isoforms. Such relative levels are subject to a plethora of parameters, ranging from differences in the protein stability due to loss or retention of the MDM2-binding site [84], expression changes during dedifferentiation as discussed above [104] or to DNA damage-induced splicing or alternative translation initiation, which have recently been reviewed by Avery-Kiejda and colleagues [114]. Titration experiments suggest that gradual changes of the alternative isoform/p53α ratio in mixed oligomers do not necessarily entail a linear relationship with transcription activities. Specifically, increasing the ratio of Δ40p53α/p53α will stabilize p53α through Δ40p53α-mediated protection from MDM2-mediated degradation already at one to two Δ40p53α monomers/tetramer and antagonize the transactivation capacity of p53α at higher ratios, generating a window of maximum canonical activities at intermediate Δ40p53α/p53α ratios [83]. However, the functions of hybrid oligomers consisting of three or more types of p53 isoforms are currently not understood.

Dominant negative effects can also be generated through subcellular mislocalization (Fig. 2). Δ40p53α, for example, is primarily located in the cytoplasm and can influence the localization of p53α by complexing it and shifting it from the nucleus to the cytoplasm [115]. More recently, protein aggregation has been understood to represent even another dominant negative mechanism acting on p53α (Fig. 2). The TAD of p53α was shown to significantly inhibit aggregation of the p53α DBD which explains why Δ40p53 possesses a higher aggregation tendency [116]. Δ133p53 aggregates much faster than p53α or p53C (p53 fragment engineered to cover amino acids 93-393) [117]. Thus, Δ133p53 could exert its dominant negative effects on canonical and non-canonical p53α functions by forming non-productive aggregates with p53α or by blocking access of p53α to the response elements of target genes [8] and to DNA replication fork structures [81].

As our understanding of p53 isoforms expands, more isoforms and corresponding biochemical activities may emerge on the surface. It is becoming increasingly clear that the complexity of p53-mediated cellular responses is finely tuned through the interplay between these isoforms. Collectively p53 isoforms exert non-binary rheostat-like functions in response to stress or during differentiation impacting on the maintenance of genome integrity, cell cycle progression, senescence, apoptosis, and tumor development. Exciting research continues to unravel the functional diversity and nuances of these isoforms, shedding light on their potential as therapeutic targets for cancer.

## The role of p53 isoforms in cancers

Accumulating evidence indicates that dysregulation of p53 isoforms co-expressed in cells may change not only cellular p53 responses, but indeed can drive oncogenesis and modify sensitivity to certain cancer treatments [2, 114, 118, 119]. Table 2 summarizes the observations made on the dysregulations of p53 isoforms in different tumor types and their clinical impact.

**Table 2:** The expression of p53 isoforms and their association with clinicopathologic outcomes in human cancers.

Isoforms	Key results	Sample size	Tumor type	Ref.
p53β	• mRNA expression is related to estrogen receptor (ER) expression in breast cancer (BRCa). *TP53* mutation status is related to cancer recurrence in the p53β-positive cohort	127	BRCa	[125]
	• mRNA expression correlated with better disease-free survival (DFS) in BRCa patients particularly in presence of oncogenic mutant p53	130		[121]
	• Protein expressed in tumor cells and tumor-infiltrating lymphocytes by immunohistochemistry (IHC) correlated with worse DFS	108		[182]
	• Highly p53β expressing patients showed enrichment of *Checkpoint Kinase 2* (*CHEK2*) sequence variants	137		[119]
	• mRNA overexpressed in colon adenoma versus non-adenoma/normal colon tissue	29	CRC	[102]
	• elevated mRNA in renal cell carcinoma (RCC) samples correlating with tumor stage; *TP53* mutation status unknown	41	RCC	[183]
	• High mRNA expression correlated with improved DFS and overall survival (OS) regardless of *TP53* mutational status RCC	266		[120]
	• high protein expression associated with marker of improved OS (mutated *Nucleophosmin* (*NPM1)*) in acute myeloid leukemia (AML) with wild-type *TP53*	68	AML	[122]
	• p53β was downregulated in AML cell lines with valproic acid (VPA) treatment and patients with high VPA sensitivity. Patient samples of a less differentiated phenotype (lower French–American–British classification) express higher levels of p53β	29		[184]
	• mRNA detected in squamous cell carcinoma of the head and neck (HNSCC), tumor adjacent tissues and normal tissues	21	HNSCC	[124]
	• mRNA and protein detected in melanoma cell lines but not melanocytes	19 cell lines	Melanoma	[129]
	• decreased p53β mRNA level in melanoma (wild-type *TP53*) associated with short OS	38		[123]
	• lower p53β/p53(α+β+γ) expression ratio associated with aggressive melanoma and worse survival	123		[185]
p53γ	• mRNA expression in mutant p53 expressing breast tumors improves DFS	127	BRCa	[125]
	• mRNA increase in uterine serous carcinoma (USC) associated with reduced DFS (2/27 samples with somatic *TP53* mutations)	79	USC	[126]
	• high protein expression associated with marker (mutated *NPM1*) of improved OS in AML with wild-type *TP53*	68	AML	[122]
	• p53γ was downregulated in AML cell lines with VPA treatment and patients with high VPA sensitivity. Patient samples with lower FAB classification express higher levels of p53γ.	29		[184]
	• mRNA detected in HNSCC, tumor adjacent tissues and normal tissues	21	HNSCC	[124]
	• plasma protein seroreactivity higher in individuals with premalignant lesions compared to healthy individuals	110	CRC	[127]
Δ40p53α	• increased mRNA in BRCa and associated with triple negative subtype; high Δ40p53/p53α ratio (> 0.7) associated with worse DFS	130	BRCa	[121]
	• mRNA overexpression decreased migration and invasion properties of transduced ER+ BRCa cell lines	54		[135]
	• high Δ40p53 mRNA levels associate with proliferation, DNA damage response (e.g. *TP53*) and stemness markers (e.g. *NANOG*) • high Δ40p53/p53 ratio promoted tumor growth, stemness features and chemoresistance (doxorubicin treatment) in tumor cell line xenotransplants in vivo	148		[130]
	• high mRNA expression correlated with better recurrence-free survival (RFS) in patients with wild-type*TP53* ovarian cancer	154, 166 *	mucinous/ serous ovarian cancer	[131, 132]
	• mRNA highly expressed in melanoma cell lines but low in melanocytes	19 cell lines	Melanoma	[129]
	• protein detected in primary and xenografted glioblastoma tissues not in non-tumor cerebral cortex	43	Glioblastoma	[128]
Δ40p53β/γ	• mRNA expression reduced in melanoma versus normal tissues	38	Melanoma	[123]
	• plasma protein seroreactivity higher in individuals with premalignant lesions (β, γ) or CRC (β) compared to healthy individuals	110	CRC	[127]
Δ133p53α	• high mRNA expression correlated with improved RFS with wild-type p53 expressing serous ovarian cancer as well as both RFS and OS in patients with mutant p53	154	Ovarian cancer	[131]
	• reduced mRNA expression in endometroid ovarian cancer compared to mucinous and serous ovarian cancer as well as normal tissue	166		[132]
	• elevated mRNA expression in high-grade serous ovarian cancer associated with improved OS and progression-free survival (PFS)	69		[186]
	• reduced mRNA expression in colon adenoma, but increased in colon carcinoma compared to non-adenoma/normal colon tissue	8	CRC	[102]
	• elevated mRNA expression correlated with poorer DFS in colorectal tumors	35		[143]
	• increased expression and Δ133p53/p53 (mRNA) ratio in Cholangiocarcinoma associated with poorer OS	48	Cholangiocarcinoma	[153]
	• lower mRNA expression in RCC with wild-type *TP53* versus normal adjacent tissue	41	RCC	[187]
	• mRNA detected in HNSCC, tumor adjacent tissues and normal tissues	21	HNSCC	[124]
	• elevated mRNA level in lung cancer versus adjacent non-cancer tissue	17	Lung cancer	[154]
	• elevated protein level in melanoma versus normal tissue	38	Melanoma	[123]
	• elevated mRNA expression and Δ133p53/p53α mRNA ratio in esophageal squamous cell carcinoma (ESCC) versus adjacent normal tissue; Δ133p53/p53α mRNA ratio in serum correlated with poor OS and PFS, in serum of resected patients also with higher recurrence rates	180	ESCC	[155]
	• promotes tumor development via increasing proliferation and inflammation	NA	Not specific	[97]
	• promotes tumor invasion and metastasis via interleukin-6 activation of JAK-STAT and RhoA-ROCK signaling	NA	Not specific	[143]
Δ133p53β/γ	• mRNA expression decreased in Human Epidermal Growth Factor Receptor 2 positive (HER2+) breast tumors, associated with *TP53* mutation status and with poorer DFS and OS	273	BRCa	[150]
	• increased cytoplasmic Δ133p53β protein contributes to a worse DFS independently of TP53 mutations	108		[182]
	• Δ133p53βs protect tumor cells from apoptosis via interacting with RhoB • high Δ133p53β mRNA level correlated with higher risk of recurrence	36	CRC	[168]
	• Δ133p53γ plasma protein seroreactivity higher in individuals with premalignant lesions or CRC compared to healthy individuals	110		[127]
	• increased mRNA expression in wild-type versus mutant *TP53* glioblastoma • **∆**122p53 expression in mice causes resistance to temozolomide treatment and oxidative stress.	89	Glioblastoma	[167]
	• increased Δ133p53β mRNA level in melanoma associated with short OS	38	Melanoma	[123]
	• *elevated mRNA level in prostate cancers with wild-type TP53 correlating with shorter* PFS	122	Prostate cancer	[152]
	• expression associated with accelerated brain metastasis; expression in cell lines increased cancer-promoting proteins on the cell surface (e.g. VEGFR) and downstream p-AKT and p-MAPK signaling	27, 21, 46, 46 *	Brain, BRCa, CRC, lung cancer, Melanoma	[188]
Δ160p53α/β	• elevated Δ160p53α protein level in melanoma compared to normal tissue	38	Melanoma	[123]
	• Δ160p53α was the most variable p53 isoform in different melanoma-derived cell lines.	27	Melanoma	[156]
Δ160p53γ	• plasma seroreactivity higher in individuals with premalignant lesions or CRC compared to healthy individuals	110	CRC	[127]

*NA* not available.

*indicates sample size for different tumor types.

### p53 isoforms influence tumor development

The transcriptional activities of p53β, p53γ, and Δ40p53α play a critical role in tumor development and progression, as they regulate gene expression through collaboration with (p53β, p53γ, Δ40p53α) and modulation of (Δ40p53α) p53α´s transcriptional activities as well as through p53α-independent gene regulation (Δ40p53α) (see Table 1). Of note, regulation of p53α-dependent activities encompasses both transcriptional transactivation as well as repression of p53α target gene expression. In line with these regulatory principles, increased expression of p53β was found to correlate with better disease-free survival (DFS) and/or overall survival (OS) in breast cancer (BRCa) (mRNA), renal cell carcinoma (RCC) (mRNA), acute myeloid leukemia (AML) (protein) and melanoma (mRNA and protein) patients [120–123] and was detected in several other tumor types [102, 124] (Table 2). Overexpression of p53β in normal fibroblasts that endogenously co-express p53 isoforms feature induction of apoptosis and cell senescence via up-regulation of genes including *BAX*, *CDKN1A* and *miR-34* in a p53α-dependent manner [102]. In cancer cell lines, namely MCF7 (BRCa) and H1299 (lung cancer), p53β enhanced p53α transcriptional activity of p53α on *CDKN1A* and/or *BAX* promoters [93]. p53γ expression correlated with better DFS and/or OS in BRCa (mRNA) and AML (protein) while the opposite result was found in uterine serous carcinoma (USC), yet for the mRNA level [122, 125, 126]. Moreover, its mRNA expression was elevated in tumor tissue of squamous cell carcinoma of the head and neck (HNSCC) but not in non-tumor control tissue. Most interestingly with regard to diagnostic applications, aiming at early detection of tumors, seroreactivity of p53 was elevated in individuals with premalignant colorectal cancer (CRC) lesions [124, 127]. Similar to p53β, p53γ increased transcriptional activity of p53α on the *BAX* promoter which possesses pro-apoptotic capacity in MCF7 cells [93]. In analogy to p53α, this canonical function most plausibly connects with the association of p53β/p53γ expression with better DFS/OS. In p53 mutant expressing BRCa, p53β and p53γ may compensate the loss of function of p53α and result in low cancer recurrence and an OS as good as that of BRCa expressing wild-type p53α [121, 125].

Δ40p53α mRNA expression levels in melanoma, glioblastoma and BRCa are higher than those in corresponding normal tissues [121, 128, 129]. It is also found to be associated with aggressive tumor growth and triple-negative BRCa with mutated *TP53* [121, 130]. Along this line, a higher Δ40p53α/p53α ratio associates with worse DFS of BRCa, however, in mucinous/serous ovarian cancer with better recurrence-free survival (RFS) [131–133]. In BRCa, a high Δ40p53α/p53α ratio was reported to increase tumor growth by promoting the stemness phenotype [96], which indeed has been linked with basal-like and triple-negative BRCa [134]. However, the same research group also discovered that in the luminal human BRCa cell lines MCF-7 and ZR75-1, Δ40p53α at basal level decreases migration and invasion mimicking the role of p53α in suppressing cellular mobility and proliferation [135]. In the same cell lines, other researchers attributed a DNA repair stimulatory role to a high Δ40p53α versus p53α level by de-repressing *RAD51* [116]. In transgenic mice, Δ40p53α collaborates with p53α to transactivate *Cdkn1a*, *Mdm2*, and *Igfbp-3* to regulate the cell cycle [136]. In lung cancer, CRC, melanoma cancer cells, Δ40p53α regulates the miR-4671-5p-SGSH axis, BAX and PIDD to control cell cycle and apoptosis [84, 137, 138]. Additionally, Δ40p53α inactivates the PKR-elF2α pathway to inhibit autophagy in lung cancer and CRC cells [139]. In summary, Δ40p53α executes canonical functions regulating expression of multiple transcriptional targets with pro- and anti-tumorigenic effects, in collaboration with p53α but in some cases also in opposition to it. The final outcome of the p53α-modulatory impact of Δ40p53α might depend on the cellular context, e.g. the *TP53* mutation status, the absolute and relative levels of Δ40p53α and p53α, cancer stemness, and finally genotoxic treatment of the cells. Underscoring the relevance of treatment, testing non-canonical functions of Δ40p53α in DNA replication revealed a dominant negative function towards co-expressed p53α in K562 leukemia cells upon exposure to the DNA cross-linking agent Mitomycin C but not in cells during unperturbed growth [81].

The alternative p53 isoform Δ133p53, in particular, impacts on signaling pathways related to tumor development. In mice Δ122p53/Δ133p53 promotes inflammatory responses often preceding the onset of tumor formation [97, 140–143]. Luciferase reporter assay demonstrated that Δ133p53 stimulates NF-κB activity following *Helicobacter pylori* infection, a significant risk factor for gastric cancer. This activation leads to the upregulation of NF-κB target genes including anti-apoptotic protein BCL-2 and pro-inflammatory factors IL-6 and IL-8 [144]. Treatment with the NF-κB inhibitor pyrrolidine dithiocarbamate resulted in the downregulation of Δ133p53 mRNA levels in MKN45 gastric cancer cells [145]. This suggests a regulatory feedback loop between Δ133p53 and NF-κB, a key regulatory factor during tumorigenesis and tumor progression via cell-intrinsic and extrinsic mechanisms ranging from cell death prevention [146], epithelial-mesenchymal transition (EMT) triggering metastasis [147], and anticancer drug resistance [148] to the regulation of tumor-associated macrophages [149]. Increased mRNA expression of the Δ133p53α isoform relative to p53α correlated with poor DFS in CRC patients. Here, the underlying mechanism was found to be tumor invasion promoted by the Δ133p53α activated JAK-STAT3 and RhoA-ROCK signaling [143]. Besides, Δ122p53/Δ133p53 facilitates tumor migration and invasion by downregulating E-cadherin and β1-integrin, upregulating *Itgb7* and *Vcam1*, or activating RhoA-ROCK signaling [97, 143, 150]. In support of a role in tumor-associated inflammatory responses, Δ133p53α activates the expression of IFN-γ signaling genes in ER+ BRCa with mutant p53 [151]. Moreover, overexpression of Δ133p53β enhances the expression of genes associated with the IFN-γ signaling pathway in prostate cancer [152].

The dominant negative effect of Δ133p53 is central to its role in tumor development and progression underscored by the fact that Δ133p53 isoforms were never expressed alone as the only p53 isoform in cancer or normal cells [102]. Δ133p53α isoforms were overexpressed in tumor tissues of cholangiocarcinoma, lung cancer, colon cancer, ovarian cancer, melanoma and esophageal squamous cell carcinoma (ESCC) [102, 123, 131, 153–155]. Elevated Δ133p53α/β versus p53α mRNA ratio was correlated with poorer OS or DFS in various cancer patients, namely in cholangiocarcinoma, ESCC, melanoma, prostate cancer [123, 152, 153, 155] (Table 2). Δ133p53α (Δ122p53α/Δ113p53α, mouse/zebrafish orthologs of human Δ133p53) dominant negatively regulates the transactivation of *MDM2, CDKN1A, PUMA, NOXA, BAX* and *miR-34a* by p53α to control cell cycle and apoptosis [97, 98, 103].

Δ160p53α was found to be upregulated in melanoma compared to non-tumor tissue [123]. As shown for Δ133p53α in cell lines [7], Δ160p53α/β can be induced by doxorubicin, cisplatin as well as etoposide in melanoma [156]. Different from the p53α-mediated transactivation of the internal *TP53* promoter in case of Δ133p53α in cell lines [7], translation of Δ160p53α is induced by oncogenic mutant p53 proteins and in this way involved in their gain of function phenotype [92] promoting tumor proliferation and migration. Importantly, Δ160p53α can be imported into the nucleus and bind chromatin [156].

Both Δ133p53 and Δ160p53, i.e. the p53 isoforms with prominent dominant negative biological effects, were shown to affect the usage of the error-free p53- and POLɩ-dependent DDT pathway triggered by co-expressed p53α [81]. Strikingly also, Δ133p53 and Δ160p53 caused fork stalling during DNA replication [81]. Such fork stalling was already noticeable under unperturbed growth conditions and already without co-expression of p53α suggesting an inhibitory effect on p53α co-factors of the DDT pathway [64, 81]. Indeed, the formation of POLɩ foci was downregulated in the presence of Δ133p53 or Δ160p53, which could be explained by the non-productive occupation of forks blocking access to replication factors such as POLɩ. Independently of the precise mechanism affecting such non-canonical function of p53α, both fork stalling and dysregulated DDT pathway usage are known to cause genomic instability and thereby tumorigenesis [89]. Moreover, loss of fork protection and resulting DNA damage are known to be sufficient to induce EMT genes and thereby invasiveness and metastasis [157].

Taken together, both canonical and non-canonical functions of alternative p53 isoforms can promote initiation and progression of cancer. Among the many factors determining the fate of a cell on the route towards malignancy, the expression levels of the p53 isoforms are essential. Evidence above demonstrates that most cancer types listed in Table 2 feature high expression levels of N-terminally truncated isoforms correlating with poorer prognosis while the C-terminally altered isoforms cause heterogenous effects.

### p53 isoforms and responsiveness to cancer therapy

Cancer therapy resistance remains a significant challenge in the management of various malignancies. Radiation therapy and chemotherapy are important components of cancer therapy, which often exert their anti-tumor effects by interfering with DNA replication processes and/or inducing DNA damage [158]. While p53α is known to be a key modulator of these processes [159], whether isoforms that are C-terminally altered or N-terminally truncated are involved in these processes and impact on cancer therapy resistance has remained underexplored.

So far, it has been reported that C-terminally altered isoforms of p53 affect the cancer therapy resistance via altered transcriptional activity. Despite lacking the OD, p53β was shown to form a complex with p53α and exhibit a preference for specific p53 response elements (e.g. *BAX* promoter) after treatment with actinomycin D, which leads to apoptosis [94]. Furthermore, the ectopic expression of p53β in melanoma cell lines augmented transcription of *CDKN1A* and *PUMA* mediated by p53α upon cisplatin treatment [129]. H1299 cells with stable transduction of p53β displayed heightened susceptibility to DNA-damaging agents including doxorubicin and camptothecin in a p53α-independent manner [80]. Upon exposure to ionizing radiation (IR), alternative splicing of p53 pre-mRNA results in the generation of p53β mRNA and protein, which is essential for the activation of genes involved in cellular senescence [160]. Similar to p53β, H1299 cells transduced with p53γ exhibited heightened sensitivity to doxorubicin and camptothecin, which correlated with increased p21 and BAX expression. However, the protein level of p53γ was found to decrease after doxorubicin treatment. Additionally, treatment with a combination of doxorubicin and dicoumarol (an NAD(P)H quinone oxidoreductase 1 inhibitor) further escalated the degradation of p53γ [80]. Regardless of these p53γ isoform-specific features regarding protein stability, both p53β and p53γ have the potential to sensitize cancer cells to therapy.

The p53α-POLι dependent DDT pathway bypasses DNA damage and confers resistance to DNA stressors such as Mitomycin C or cisplatin [36, 64, 81, 161]. Singly expressed p53β and p53γ failed to mediate this pathway after treatment, and also did not exert a dominant negative effect on co-expressed p53α in DNA fiber assays [81]. From this, a major role of p53β/p53γ in chemotherapy resistance via usage of such a DDT pathway is unlikely. Taken together, C-terminally altered isoforms seem to sensitize cancer cells via transcriptional activities without additional influence of canonical functions in DNA replication. Given that multiple DNA repair factors bind to the C-terminus of p53α, it will be interesting to see whether canonical functions in DNA repair are affected, such as observed for Δ40p53 [87].

N-terminally truncated p53 isoforms were reported to influence resistance to cancer therapy via a dominant negative effect. In melanoma cell lines, Δ40p53α impedes cisplatin-induced p53α-dependent transcriptional activation of the *PUMA* and *CDKN1A* promoters [129]. Along this line, Δ40p53α antagonized p53α-mediated transcription of *BAX*, *NOXA*, *PUMA*, and *CDKN1A* post-treatment with doxorubicin but not with cisplatin reducing the fraction of cells arrested in G1-phase and undergoing apoptosis [87]. When exposed to 5-fluorouracil, the presence of Δ40p53 similarly hindered the canonical transcriptional activity of p53α [162]. Consistently, a high Δ40p53α/p53α ratio was shown not only to promote MCF7 cell growth and stemness features but also resistance to doxorubicin treatment after transplantation into immunodeficient mice [130].

DNA repair-related functions of p53α are also modulated by overexpression of Δ40p53α facilitating the transcription of RAD51 otherwise repressed by p53α [87, 163]. However, such result might also be explained at least in part by the Δ40p53α-induced cell cycle shift, since RAD51 is expressed during S- and G2-phase in the cell cycle [164]. Moreover, in BRCa cell lines, doxorubicin treatment led to the formation of nuclear p53α-RAD51-BRCA1 but not to Δ40p53α-RAD51-BRCA1 complexes [87], suggesting that unlike p53α, Δ40p53α is not involved in the non-canonical function of the fidelity control of homologous recombination [56, 74, 165, 166]. Accordingly, aberrant DNA repair processes are predicted to become increasingly de-restricted with increasing Δ40p53α/p53α ratio, which however, needs to be substantiated experimentally.

During replication stress caused by DNA damage-inducing agents, the dominant negative effect of the N-terminally truncated isoforms Δ133/160p53α affects the p53α-POLι dependent DDT pathway which facilitates the successful bypass of DNA damage and prevents the replication fork from collapsing [81]. As this pathway has been linked with resistance to Mitomycin C and to cisplatin [64, 161], Δ133p53α and Δ160p53α are predicted to sensitize cancer cells to DNA crosslinking agents via this transcription-independent mechanism. On the other hand, Δ133p53α affects canonical p53 functions in the DNA damage response post-treatment with doxorubicin by forming a complex with p53α, antagonizing induction of the G1 arrest (via repressing *CDKN1A* transactivation) and of apoptosis (de-repressing transcription of *BCL-2*). Of note, Δ133p53α is upregulated by p53α-dependent transcriptional transactivation at the internal promoter in response to a low dose of this DNA intercalating agent triggering such a negative feedback loop [7]. Furthermore, Δ113p53 (zebrafish ortholog of human Δ133p53) collaborates with p73 to promote DNA double-strand repair via *rad51*, *Lig4* and *rad52* upregulation. In 10.1 cells (mouse p53-null fibroblast cell line), Δ122p53β (murine Δ133p53β isoform) reduced sensitivities to temozolomide and tert-butyl hydroperoxide, two treatments inducing base damage [167]. In CRC cells, Δ133p53β hindered camptothecin, i.e. topoisomerase I inhibitor-induced apoptosis by binding to RhoB [168]. Altogether, N-terminally truncated p53 isoforms have the potential to cause chemoresistance of cancer cells by both canonical and non-canonical functions. The net outcome of these different responses to genotoxic drugs might depend not only on the cellular context and on p53 isoform levels but also on the type and severity of DNA damage.

p53 isoforms play crucial roles in tumor development and cancer therapy resistance. Understanding the roles and mechanisms of these p53 isoforms is essential for developing effective strategies to combat cancer. Dominant negative effects on transcriptional activities and on transcription-independent DNA replication regulatory activities are key mechanisms through which p53 isoforms exhibit their biological functions and influence tumor development and cancer therapy resistance. Hence, integrating such knowledge from different cancer entities and treatment regimens will help to develop refined strategies to overcome therapy resistance to cancer treatment.

## Conclusion

The study of p53 isoforms has provided significant insights into the complex biological roles of the p53 protein in both normal cellular functions and cancer development. The diverse isoforms of p53 exhibit canonical functions in transcriptionally transactivating or repressing target genes, impacting on cell cycle control, apoptosis induction and DNA repair, in particular. Aside from these canonical functions, non-canonical, i.e. transcription-independent functions of these isoforms in DNA repair and replication have been discovered more recently. In a concerted action, integrating cooperative, antagonistic and independent activities of the different isoforms, these biological functions modulate tumor suppressor activities and influence the sensitivity of cancer cells to therapies.

Aside from the structural diversity of p53 isoforms abnormal expression or dysregulation of p53 isoforms have been implicated in various types of cancers, contributing to tumor progression, metastasis, and drug resistance. It is known that the level of p53α plays a critical role in differentially activating different gene sets [169], and can rapidly be induced by genotoxic treatment impacting on the cellular program [170]. Intriguingly, it has also been observed that the level of p53 determines the DDT pathway chosen to bypass replication impediments (L.W., personal communication). Given that in Guo et al. Δ133p53α and Δ160p53α were found to abrogate such p53α functions, a change in cellular levels of p53α, Δ133p53α and/or Δ160p53α will affect the pathway choice by mixed oligomer formation or competition for DDT pathway components [81]. Thus, both for canonical as well as noncanonical functions the levels of p53 isoforms are relevant, fine-tuning the outcome of activating specific gene sets and specific DDT pathways, respectively.

However, despite all these advances further research is warranted to elucidate the precise molecular mechanisms by which p53 isoforms exert their effects and to reveal their potential as diagnostic and prognostic biomarkers. First, non-canonical functions of alternative p53 isoforms in DNA repair are underexplored, though structural differences between p53 isoforms affecting the interaction sites with crucial DNA repair factors like POLβ [33], RAD51 [56, 74, 165, 166] or BRCA1 [87] must impact on base excision repair and homologous recombination, respectively. Second, it remains to be determined in how far non-canonical functions of alternative isoforms in DNA replication and repair modify stemness features/EMT of cancer cells and thereby invasiveness, metastasis and chemoresistance. Third, results from functional assays have become valuable tools for the classification of germline variants of *TP53* with regard to their pathogenicity [171–173]. Yet, the impact of alternative splicing as well as of alternative transcription or translation start sites have been largely ignored. Understanding the functional diversity and interplay of p53 isoforms may pave the way for personalized approaches towards cancer prevention and the development of innovative therapies.
